# A smart deoxyribozyme-based fluorescent sensor for in vitro detection of androgen receptor mRNA

**DOI:** 10.3762/bjoc.16.100

**Published:** 2020-05-27

**Authors:** Ekaterina Alexandrovna Bryushkova, Erik Rafikovich Gandalipov, Julia Victorovna Nuzhina

**Affiliations:** 1 Department of Molecular Biology, Lomonosov Moscow State University, Lenin Hills 1/12, Moscow, 119991, Russian Federation; 2 Laboratory of Solution Chemistry of Advanced Materials and Technologies, ITMO University, Lomonosova 9, St. Petersburg, 197101, Russian Federation

**Keywords:** androgen receptor, 10–23 deoxyribozyme, nucleic acid sensor, malachite green aptamer, RNA cleavage

## Abstract

Nowadays a variety of biosensors are widely used in different fields, including biomedical diagnostics and self-testing. Nucleic acid-based biosensors are typically applied to detect another nucleic acid, proteins, ions, and several other types of compounds. It is most promising to develop simple and effective biosensors for the use in situations where traditional methods are not available due to their complexity and laboriousness. In this project, a novel smart deoxyribozyme-based fluorescent sensor for the detection of androgen receptor mRNA was developed. It consists of several functional modules including two deoxyribozymes 10–23, an RNA-dependent split malachite green aptamer, and an oligonucleotide platform. Deoxyribozymes specifically release a 27-nucleotide RNA fragment that is readily available for the interaction with the aptamer module. This solves a problem of secondary structure in hybridization with the target sequence of full-length mRNA. It was shown that within 24 hours the proposed sensor specifically recognized both a synthetic 60-nucleotide RNA fragment (LOD is 1.4 nM of RNA fragment at 37 °C) and a full-sized mRNA molecule of the androgen receptor. The constructed sensor is easy to use, has high efficiency and selectivity for the RNA target, and can be reconstructed for the detection of various nucleic acid sequences due to its modular structure. Thus, similar biosensors may be useful for the differential diagnosis.

## Introduction

The fast and precise diagnostics of diseases are one of the key factors that allow choosing the most effective method of treatment. Disease markers can be found at a few different levels, including DNA, RNA, proteins, and small molecule metabolites. Today, along with other methods in clinical diagnosis biosensors are ubiquitously used in the biomedical field.

The first prototype of a biosensor was invented by Leland Clark and Champ Lyons in 1962 as an amperometric Clark electrode, covered by immobilizing enzyme glucose oxidase, for the detection of glucose [1]. A biosensor means a small molecular device that traditionally consists of a bioreceptor (enzyme, cell, aptamer, oligonucleotide, antibody, and other) for the specific recognition of the target molecule and a transducer which effectively converts the biochemical signal produced by the bioreceptor into a physically detectable and quantifiable signal [2]. The main characteristics of biosensors include selectivity, sensitivity (limit of detection or the minimum amount of analyte that can be detected), and stability. Reproducibility and linearity are also very important as well as costs and ease of manufacturing each component of the biosensor.

Unlike proteins or antibodies the nucleic acid-based biosensors (NAs) can be easily commercially synthesized, they are smaller, more stable, and can be repeatedly used without losing their binding capability [3] with sensitivities in the range from 1 mM for cocaine to 10 pM for Hg^2+^, but for many targets it averages in the 1–10 nM range [4].

Although the diversity of NAs is very large, they can be divided into DNA-based biosensors, for example, molecular beacon or TaqMan probes, deoxyribozyme-based biosensors [5], and aptamer-based biosensors [3]. The detection mainly relies on a specific hybridization between a well-known target fragment and the biosensor strands. To increase selectivity NAs are also created as binary or split construction. Due to the simplicity, the DNA hybridization technique is more frequently used in diagnostic laboratories than the direct sequencing method [6].

A serious problem for the detection of full-sized nucleic acids is the secondary structure, which interrupts the access of sensors to the binding site. For single-stranded RNA this problem may be partially solved by including additional substrate-delivering strands into the biosensor [7]. However, in general, the problem of accessibility of the target nucleic acid site is still the main disadvantage of using NAs in this field. In this project, we demonstrate an experimental model of smart deoxyribozyme-based fluorescent sensor (SDFS), designed for the quick and efficient verification of human androgen receptor (AR) mRNA.

The AR (alternative name NR3C4) belongs to the steroid nuclear receptor superfamily, capable of being activated after a direct interaction with nuclear DNA and works as a transcription factor [8]. Among the target genes of the AR are genes encoding proteins involved in intracellular signal transmission, proliferation, as well as differentiation and apoptosis [9]. An increase of AR mRNA expression or enhancing their sensitivity to the corresponding ligands may lead to the manifestation of androgenetic alopecia [10], adulthood acne or hirsutism [11], or even to impaired fat metabolism, muscle atrophy, and other metabolic disorders [12]. Thus, the AR mRNA could be considered as an important diagnostic marker in various pathologies [13].

Laboratory diagnostic methods may easily detect systemic hyperandrogenism, however, the measurement of local androgens changes or AR expression is still a serious problem and requires the development of additional test systems. Therefore, we have chosen AR mRNA as a target for our sensor. The design of SDFS included a number of functional advantages over most known biosensors and has the potential to solve the problems mentioned above.

## Results and Discussion

The human AR gene is located on the X chromosome at Xq11_12 and is encoded by eight exons. The first exon contains polymorphic CAG microsatellite repeats and codes a variable length of the N-terminal domain (NTD). In vitro studies demonstrated that the progressive expansion of the length of the CAG repeat in NTD decreased its transactivation function. Exons 2 and 3 code the central DNA-binding domain and exons 4 to 8 code the C-terminal ligand-binding domain [14–15]. There are currently described 18 alternative splice variants of AR mRNA – AR-FL, AR45, AR-23, AR V1-V14 and AR-8. Most of them encode small-sized or functionally inactive proteins [16]. However, all translated isoforms of the AR protein contain NTD, which is critical for AR function. This is the main reason to target our SDFS on the sequence inside the first exon. Although splice variants of AR-FL (full-size molecule), AR-V7, and AR-V9 [17–18] are the most important ones from a clinical point of view, we analyzed all nucleotide sequences of the translated AR mRNAs from open databases (Supporting Information File 1, Table S1 and Figure S1).

Based on the results of the bioinformatics analysis, an RNA sequence of 60 nucleotides (nt) was selected as a target (Table S2, 60-AR_RNA). This sequence was located in the conserved exon 1, position 1–2140 nt. The specificity of 60-AR_RNA for the human androgen receptor was further verified using the BLAST algorithm (https://blast.ncbi.nlm.nih.gov/Blast.cgi).

**Figure 1:**
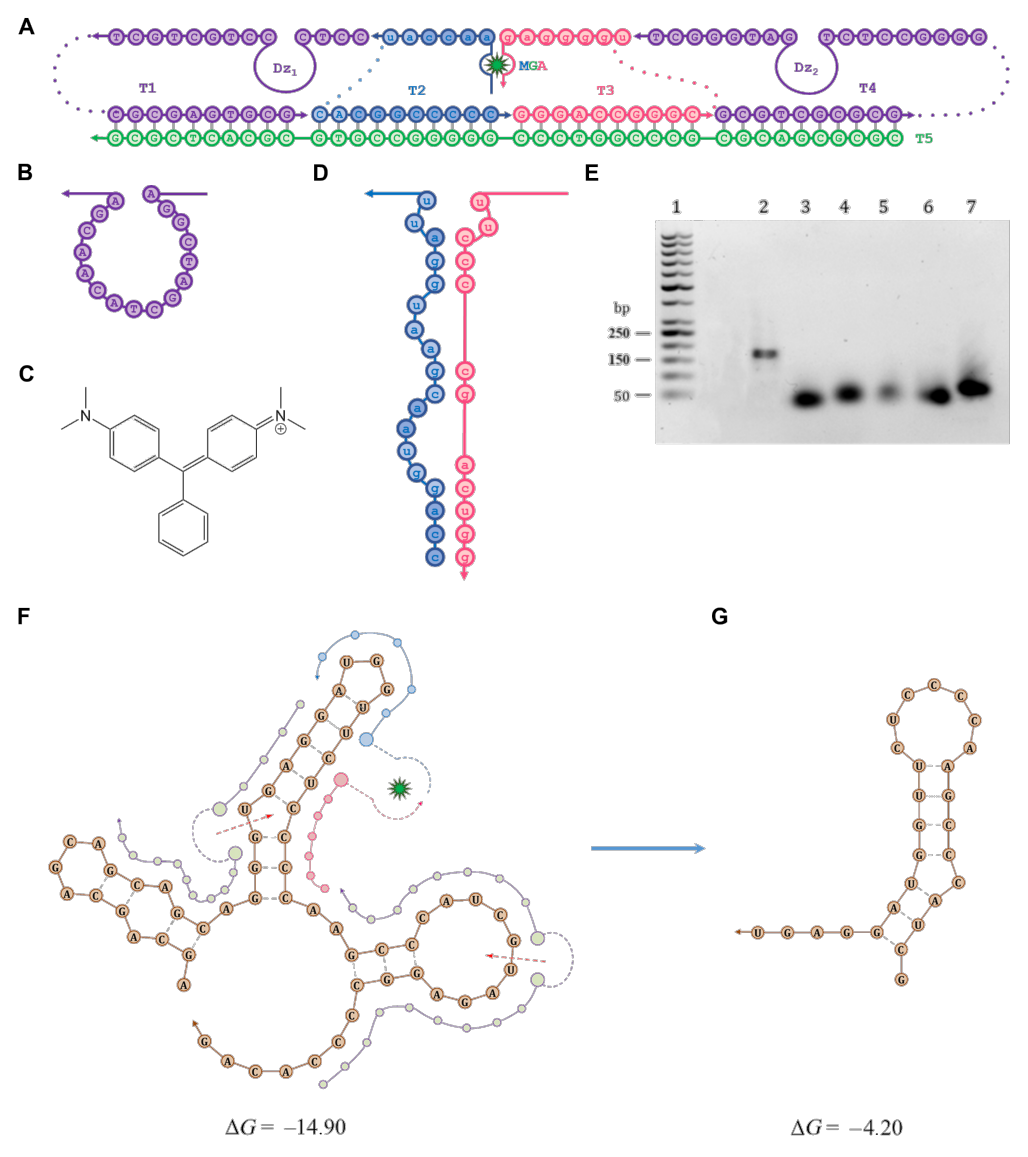
SDFS main components and its work model. A) Schematic representation of the SDFS structure. Dotted lines indicate hexaethylene glycol linkers in T1 and T4, and polythymidines linker in T2 and T3; Dz1 and Dz2 – deoxyribozyme sequences. B) The detailed structure of the Dz catalytic core cited by Silverman 2016 [19]; the green star indicates a malachite green dye molecule. C) Malachite green dye chemical structure. D) Detailed malachite green split aptamer structure cited by Kolpaschikov 2005 [20]. E) Confirmation of SDFS assembly by using agarose gel electrophoresis; lane 1: DNA ladder; lane 2: assembled SDFS; lanes 3–7: strands T1–T5, respectively. F) An initial 60-nt fragment of AR mRNA (60-AR_RNA); color lines indicate the possible orientation of the SDFS chains around the RNA structure; red arrows with dotted loops indicate the assumed cleavage sites. G) A short 27-nt fragment resulting from the 60-AR_RNA cleavage. Prediction of the RNA secondary structure with the folding energy before and after cleavage was performed by MFold open source [21].

We proposed the model of SDFS (Figure 1A) developed in our laboratory, which was based on the principles of Holliday junction and consisted of several functional parts: (i) two catalytically active deoxyribozymes 10–23 (Figure 1B) with different lengths of RNA-binding sites (Dz1 9 nt right/4 nt left and Dz2 9 nt right/8 nt left), which recognize 60-AR_RNA on both sides around the site of aptamer binding, (ii) split malachite green aptamer (Figure 1C), and (iii) an oligonucleotide platform (Figure 1A, T5 strand), that combined the components into a single structure. The T1–T4 strands were connected with the platform by polythymidine or hexaethylene glycol linkers, which provided flexibility of SDFS. All nucleotide sequences are presented in Table S2 of Supporting Information File 1. A calculation of the SDFS stability at physiological conditions was performed using the Nucleic Acid Package open service (http://www.nupack.org/).

For our sensor, a malachite green aptamer (MGA) modified by Kolpashchikov [20] was chosen as a detector. Historically, the MGA was obtained by in vitro selection from pools of random sequence molecules, also known as SELEX (systematic evolution of ligands by exponential enrichment), and was a holistic harpin [22]. In further work MGA was separated into two strands, and nucleic acid binding arms were added to each strand, allowing MGA to target a sequence of interest. Furthermore, the GAGA loop was removed and stems I and II were shortened to three and to four base pairs, respectively, in order to diminish the association of RNA strands in solution in the absence of a target [20].

In the SDFS structure split MGA has two RNA-binding sequences, which are complementary to the target 60-AR_RNA, and connected with T2 and T3 strands through polythymidine linkers (Figure 1A). The MGA strands can hybridize with each other and attach a molecule of malachite green dye (Figure 1C and D) only in the presence of target mRNA. It provided the high specificity of SDFS. After being enclosed inside a hybridized MGA, malachite green dye produces a signal (648 nm) after excitation on 610 nm, which is easily detectable by a fluorescence spectrophotometer. The complete SDFS was assembled by annealing the individual component strands (T1–T5) to each other. The efficiency of the SDFS assembling was evaluated using agarose gel electrophoresis (Figure 1E). The single band proved that in the chosen conditions there was a complete hybridization of all 5 strands into an integrated SDFS complex.

After hybridization between SDFS and 60-AR_RNA we expected to see a specific cleavage of target mRNA by Dz1 and Dz2, which are parts of T1 and T4 strands, respectively, that leads to the release of a short 27-nt fragment from 60-AR_RNA (Figure 1F and G) near the MGA. Due to this a significant increase in fluorescence signal at the presence of malachite green was expected owing to the fact that 27-nt fragment has steric and thermodynamic benefits to interact with MGA compared to 60-AR_RNA, or especially full-length AR mRNA.

Due to the close proximity of “GU” cleavage sites in the selected 60-AR_RNA fragment, we decided to use asymmetric Dz1 design with an extremely short left RNA-binding part (4 nt). In cleavage tests with the synthetic 27-nt and 46-nt RNA fragments it was shown that for Dz 10–23 an optimal length of RNA-binding parts were 7-nt right/7-nt left [23] and should not exceed 10 nt [24]. Given that the cleavage efficiency of Dz 10–23 with both asymmetric and short RNA-binding part on 60 nt was not previously evaluated, we performed a preliminary cleavage assay for the free Dz1 and Dz2 as well as for the assembled SDFS (Figure S2 in Supporting Information File 1). We demonstrated that at the presence of 2 mM MgCl_2_ all three catalytically active molecules started to cleave 60-AR_RNA at 37 °C already in 6 hours.

**Figure 2:**
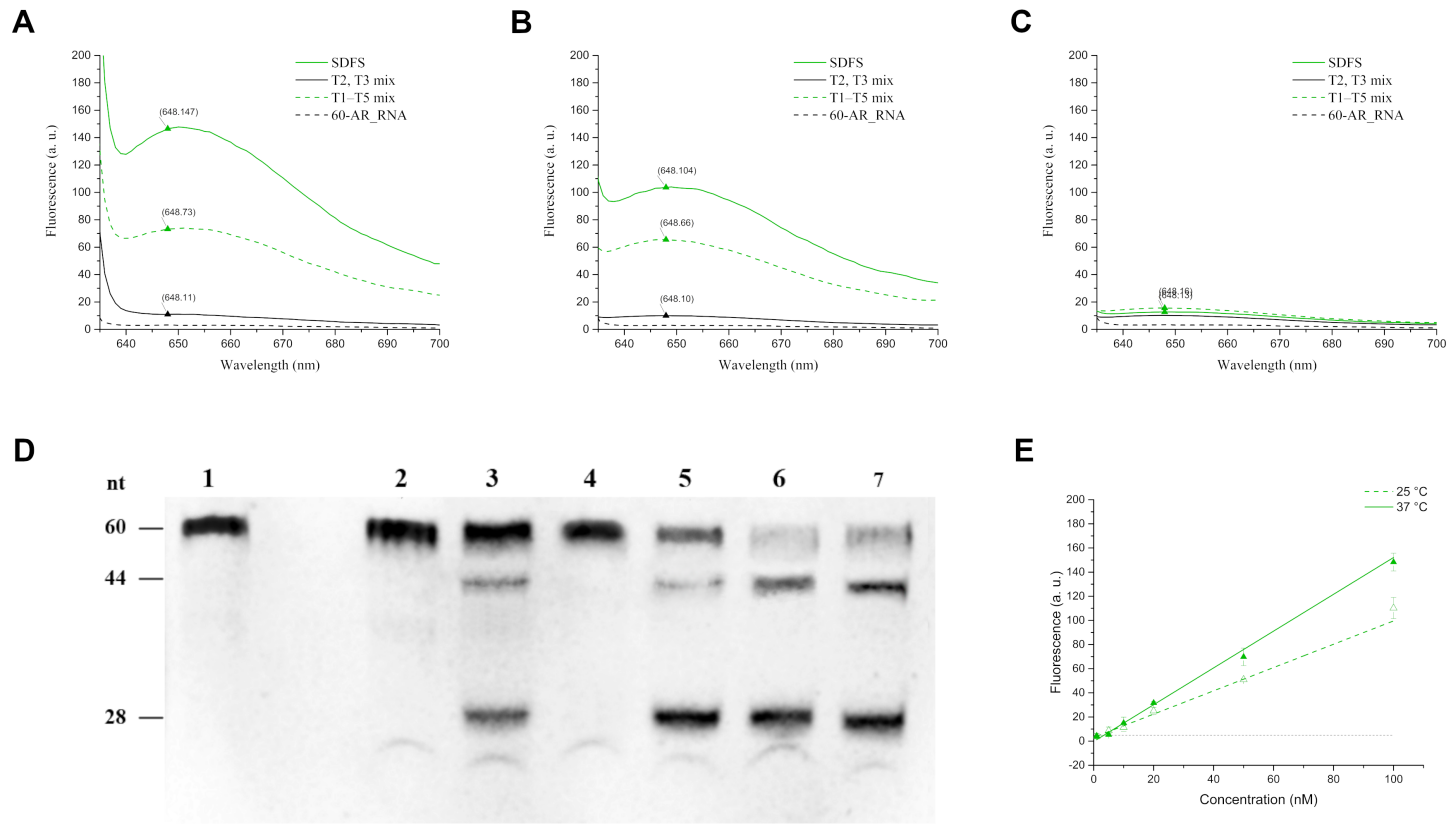
The SDFS functional activity. A–C) Emission spectra of the assembled SDFS (green line), T1–T5 chains mix without annealing (green dotted line), MGA sample containing free T2 and T3 chains (black dotted line), and a control sample with 60-AR_RNA (black line) after 24 h of incubation at 37 °C, 25 °C, and 4 °C for A), B), and C), respectively. D) Denaturing PAGE assessment of cleavage of samples containing 60-FAM-AR_RNA and different agents after 6 h of incubation; lanes 1 and 2: 60-FAM-AR_RNA incubated for 0 and 6 hours, respectively; lane 3: 60-FAM-AR_RNA and individual strands T1 and T4; lane 4: 60-FAM-AR_RNA, T2 and T3; lane 5: 60-FAM-AR_RNA with mix of T1–T5 strands; lanes 6 and 7: 60-FAM-AR_RNA with assembled SDFS in the absence or presence of malachite green, respectively. E) Approximated limit of detection for SDFS at 37 °C (green line) and 25 °C (green dotted line). Each dot is an average of triplicate values of fluorescence with standard deviations for samples with different concentrations of 60-AR_RNA. A black dashed line indicates the threshold fluorescence value of the buffer with malachite green dye and 60-AR_RNA.

To estimate the functional activity of the SDFS we simultaneously evaluated the biosensor fluorescence intensity and the efficiency of the target RNA cleavage in the presence of SDFS under the same conditions. The complete assembled SDFS (100 nM), mix of Т1–Т5 (100 nM of each strand) without annealing and mix of Т2–Т3 (100 nM for each chain) with integrated MGA, also without annealing, were incubated for 6 h or 24 h at 37 °C, 25 °C, and 4 °C with 60-AR_RNA (fluorescence assay) or for 6 h at 37 °C with FAM-60-AR_RNA (cleavage assay). The concentration of the malachite green dye was 25 μM. After incubation fluorescence measurement was conducted using fluorescence spectrophotometry (610/648 nm) (Figure 2A, B, and C) and the cleavage was verified by denaturing PAGE (Figure 2D).

According to the densitometry analysis of PAGE, after 6 h of incubation at 37 °C around 90% of FAM-60-AR_RNA were cleaved by one or both deoxyribozymes in the composition of complete SDFS, and 70% were cleaved by the mixture of separate T1–T5 strands.

In the fluorescence study several temperature conditions were chosen as optimal parameters for deoxyribozyme (37 °C) and MGA (4 °C) activity, calculated by NuPACK. The temperature of 25 °C was chosen as a compromise temperature. The observed results showed that after 24 h of incubation at 37 °C and 1 h of cooling at 4 °C, the effectiveness of SDFS was highest among the tested parameters. Cooling was an essential part of sample preparation still 4 °C was an optimal temperature for MGA to assemble into the complex with the dye molecule. In such conditions the increase of fluorescence signal was 13 times higher than the signal demonstrated by individual MGA strands and 5 times higher than the signal given by unassembled SDFS strains (Figure 2A). At 25 °C, SDFS gave more unassuming results after 24 h of incubation and 1 h of cooling at 4 °C, demonstrating only 10 times increased fluorescent signal in comparison with individual MGA strands (Figure 2B). Finally, there was no significant fluorescence increase observed after incubation at 4 °C for 24 h. These results were consistent with our hypothesis on facilitating the ease of access to target 60-AR_RNA by cutting a short 27-nt fragment from the full target sequence by deoxyribozymes, thus increasing the local concentration of this fragment near to the sensing part of SDFS (Figure 2D, lane 6). As follows from Figure 2D (lanes 6 and 7), the presence of malachite green dye did not affect the catalytic activity of SDFS.

Selectivity of SDFS cleavage activity in relation to 60-AR_RNA was tested with two random RNA sequences (Supporting Information File 1, Table S2, 62-RNA and 46-RNA) with lengths or secondary structures similar to 60-AR_RNA. Both of these sequences contained a “GU” site for deoxyribozyme 10–23 cleavage, but were not complementary to any of the components of the SDFS. Figure S4 (Supporting Information File 1) demonstrates that non-specific cleavage of both random RNA targets in the presence of SDFS did not occur.

Furthermore, even after 6 h of incubation at 37 °C the intensity of the fluorescent signal in the sample containing the assembled SDFS with 60-AR_RNA was 10 times higher than the signal demonstrated by individual MGA strands, and 5 times higher than the signal given by unassembled SDFS strands (Figure S3 in Supporting Information File 1). However, after 6 h of incubation at 4 °C or 25 °C, SDFS did not demonstrate any significant fluorescence signal in comparison with individual MGA strands.

The next step was to assess the limit of detection (LOD) of SDFS. For this experiment, samples containing 100 nM of annealed SDFS and 25 μM of malachite green dye in the same buffer were incubated with different amounts of 60-AR_RNA (1 nM, 5 nM, 10 nM, 20 nM, 50 nM, and 100 nM) for the above-mentioned times and temperatures. According to the obtained data, the LODs of SDFS were 1.4 nM for 24 h at 37 °C, 3.5 nM for 24 h at 25 °C (Figure 2E), and 1.6 nM for 6 h at 37 °C (Figure S3B in Supporting Information File 1).

These data proposed our biosensor as a suitable agent for the rapid detection (but only at a temperature that is optimal for cleavage) or detection at room temperature (but for longer periods of incubation).

Despite the fact that MGA has long been known, there is still insufficient experimental data on the sensitivity of it. It was mentioned that single harpin MGA increased the fluorescence of the malachite green dye by more than 2000-fold [25] upon binding. For the split MGA Kolpashchikov showed that 2 µM of a 14-nt DNA analyte could be detected using MGA at the same concentration (room temperature, 1 mM MgCl_2_) [20]. He demonstrated that the addition of 2 µM 14-nt analyte increased the fluorescence intensity by about 20 times, but the limit of detection was not estimated. Compared to the known LODs for RNA-targeted biosensors, the sensitivity of SDFS was at least not lower to Spinach aptamer (LOD is 1.9–5.3 nM in similar conditions) and a little bit less than Dapoxyl aptamer (LOD is 0.44 nM). At the same time, none of the two aptamers mentioned above was tested on full-length mRNA [26].

**Figure 3:**
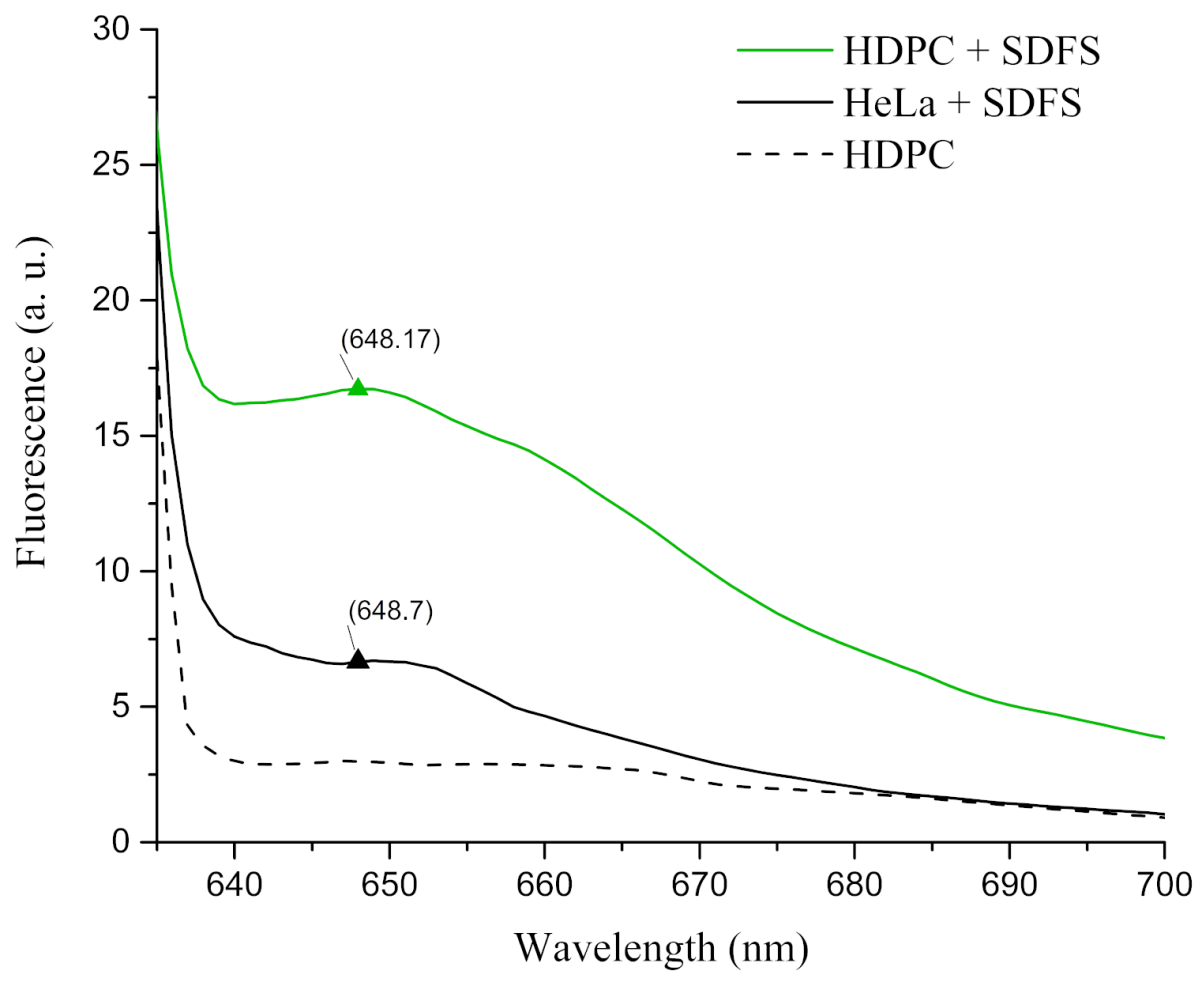
Emission spectra of SDFS activity on total cellular RNA. The green line represents a spectrum of SDFS incubated with total HDPC RNA. The black line is a spectrum of SDFS incubated with total HeLa cells RNA. The black dotted line is a spectrum of total HDPC RNA incubated in the presence of malachite green dye but without SDFS.

To investigate the ability of the proposed SDFS to selectively detect a full-length AR RNA we performed experiments on total RNA that was extracted from human dermal papilla cells (HDPC) obtained from the cell culture collection of the Koltsov Institute of Developmental Biology of the Russian Academy of Sciences (Moscow, Russia). These cells demonstrated a high level of AR mRNA expression (Figure S5, Supporting Information File 1) and by that were considered as a good model for our study. Extracted total RNA of HDPC was added to the buffer containing SDFS (100 nM) and malachite green dye (25 μM) to make a concentration of 10 μg RNA in a 50 μL sample. Then, the samples underwent incubation at 37 °C for 24 h followed by cooling at 4 °C for 1 h. The fluorescence signal of the samples was measured as it was done for the 60-AR_RNA samples. As a negative control samples containing total RNA from HeLa cells, which referred to be steroid receptor negative [27], with SDFS and malachite green dye were incubated and measured as well (Figure 3).

A comparison of the fluorescence data presented above showed that the signal from samples containing HDPC total RNA incubated with SDFS were significantly higher than the signal from the same amount of HDPC total RNA without SDFS. Moreover, the sample of medium level AR expressing HeLa cells total RNA demonstrated a two times lower signal than it was observed for HDPC cells total RNA. Thereby the obtained fluorescence spectra proved the effectiveness of the proposed full-length RNA detection mechanism.

## Conclusion

This work describes the development of a modular deoxyribose-based biosensor for the detection of androgen receptor mRNA expression. Due to cooperative work of both Dz1 and Dz2 and malachite green split aptamer combined in the single structure, SDFS allows an increase in the local concentration of short 27-nt fragments of the target mRNA near the MGA. This facilitates the access and hybridisation between the MAG and target sequence of AR mRNA. Thus, we may solve the problem of the secondary structure of RNA targets, which complicates the interaction with the biosensor and impedes their widespread use. Another advantage of our sensor is the ability to easily change different oligonucleotide sequences depending on the experimental design. Since the construct is assembled by the researcher immediately before work, it is always possible to have several different functional modules (aptamer sequences, deoxyribozymes with different lengths of RNA-binding sequences, and others) for the current task optimization. Proposed SDFS design used malachite green dye, which is easily available and cheap, but today it is not too often applied for the detection of nucleic acids. We showed that SDFS can be used to efficiently detect at least 60 nt AR mRNA fragments both at 37 °C and at room temperature (25 °C) with different sensitivity and incubation times. Thus, by varying the test setup time and the sensor/target ratio, it is possible to achieve design optimization for various tasks, and the ability to recognize full-length mRNA makes our sensor promising for further development.

The next step in this work will be the optimization of the SDFS for the detection of AR mRNA into a biological material.

## Supporting Information

File 1 Detailed experimental description.
