# Supplementary material for: A smart deoxyribozyme-based fluorescent sensor for in vitro detection of androgen receptor mRNA
Source: Beilstein J Org Chem. 2020 May 27;16:1135–41. doi: 10.3762/bjoc.16.100 (PMC7277777; doi:10.3762/bjoc.16.100)
Supplement: File 1 — Detailed experimental description. [file Beilstein_J_Org_Chem-16-1135-s001.pdf]

BEILSTEIN JOURNAL OF ORGANIC CHEMISTRY

Supporting Information

for

A smart deoxyribozyme-based fluorescent sensor for in vitro detection of androgen receptor mRNA

Ekaterina A. Bryushkova, Erik R. Gandalipov and Julia V. Nuzhina

Beilstein J. Org. Chem. **2020,** *16,* 1135–1141. doi:10.3762/bjoc.16.100

Detailed experimental description

License and Terms: This is a supporting information file under the terms of the Creative Commons Attribution License (http://creativecommons.org/licenses/by/4.0). Please note that the reuse, redistribution and reproduction in particular requires that the authors and source are credited.
The license is subject to the *Beilstein Journal of Organic Chemistry* terms and conditions: (https://www.beilstein-journals.org/bjoc)

Table of contents

1. Reagents and cell cultures

2. Bioinformatics analysis of AR mRNA transcripts

3. Oligonucleotides used in the study

4. Assembling of SDFS

5. Cleavage of FAM-60-AR_RNA assay

6. Fluorescence of SDFS in presence of 60-AR_RNA measurements

7. Limit of 60-AR_RNA detection for SDFS

8. Selectivity of SDFS

9. Total RNA extraction

10. Fluorescence of SDFS in presence of HDPC total RNA measurements

1. *Reagents and cell cultures*

DNAse/RNAse-free water was purchased from Thermo Fisher Scientific, Inc. (Pittsburgh, PA, USA) and used for preparation of all the stock solutions of oligonucleotides and buffers. All other oligonucleotides, including fluorescein-labelled RNA (see Table S1 for sequences) were purchased from DNA Synthesis (Moscow, Russian Federation). The oligonucleotides were dissolved as 100 µM solution and stored at −20 °C.

Malachite green oxalate salt was purchased from Sigma-Aldrcich (St. Louis, Missouri, USA). Other chemicals, such as KCl, NaCl, 4-(2-hydroxyethyl)-1-piperazineethanesulfonic acid (HEPES), $MgCl_2$, urea, acrylamide, *N*,*N*'-methylenebisacrylamide, boric acid, ammonium persulfate (APS), tris(hydroxymethyl)aminomethane (Tris), tetramethylethylenediamine (TEMED), agarose for molecular biology, and ethylenediaminetetraacetic acid (EDTA) were purchased from VWR Life Science AMRESCO (Radnor, PA, USA) and SYBR™ Gold Nucleic Acid Gel Stain from Invitrogen Corp. (Carlsbad, California, USA).

The human dermal papilla cells (HDPC) with high AR expression were obtained from the cell culture collection of Koltzov Institute of Developmental Biology of the Russian Academy of Sciences (Moscow,

Russia). Human cervix adenocarcinoma cells (HeLa) were purchased from Biolot (Moscow, Russia). Both cell lines were cultivated in DMEM from Biolot (Moscow, Russia) with 10% fetal bovine serum (FBS) from Thermo Fisher Scientific, Inc. (Pittsburgh, PA, USA) and 50 µg/mL of gentamicin from Biolot (Moscow, Russia) at 37 °C, 5% CO_2.

ExtractRNA reagent for total RNA extraction was purchased from Evrogen (Moscow, Russia). For AR gene amplification qPCRmix-HS and MMLV reverse transcriptase with corresponding buffers were also purchased from Evrogen (Moscow, Russia). BrightMAX™ 25–700 bp DNA ladder was purchased from Canvax (Córdoba, Spain).

2. *Bioinformatics analysis of AR mRNA transcripts*

The list of the most clinically important alternatively spliced AR transcripts with known nucleotide sequences has been compiled from Wach et al, 2020 [1] (Table S1). Based on this list, we used the sequences of spliced variants available in the NCBI RefSeq (https://www.ncbi.nlm.nih.gov/refseq/) and GenBank databases (https://www.ncbi. nlm.nih.gov/nuccore/). To identify mRNA regions that were homologous to the largest number of alternative transcripts, the multiple alignment method was used, specifically the ClustalW algorithm (http://www.clustal.org/clustal2/) as a part of the Unipro UGENE software (UniPro, Russia) (Fig. S1), and the content of single nucleotide polymorphisms was analyzed using the NCBI dbSNP database (https://www.ncbi.nlm.nih.gov/snp/).

The sequence NM_000044.6 (NCBI Reference Sequence) was used to select the most suitable target site of AR mRNA for our study (60-AR_RNA). Then, bioinformatics analysis of the human AR mRNA was conducted using several evaluation criteria: (i) the target sequence should be found in the largest number of RNA transcripts of the AR gene, (ii) the target sequence should be specific only for the mRNA of the AR gene, (iii) the target sequence should possess low percentage of variability in single nucleotide polymorphisms, and (iv) the presence of one or more deoxyribozyme-specific “gu” cleavage sites.

Table S1: AR transcripts, used for the selection of potential mRNA target region for SDFS.

Variant names	Transcription features	Source
AR-FL (AR1)	Ligand-dependent	NM_000044.6 (NCBI Reference Sequence)
AR45 (AR2)	Regulated	NM_001011645.3 (NCBI Reference Sequence)
AR-V7 (AR3)	Constitutive	NM_001348061.1 (NCBI Reference Sequence)
AR-V1 (AR4)	Regulated	NM_001348063.1 (NCBI Reference Sequence)
AR-V3	Constitutive	sequence is not available
AR-V4	Constitutive	sequence is not available
AR-V9	Regulated	sequence is not available
AR-V12 (AR567es)	Constitutive	GU208210.1 (GenBank)

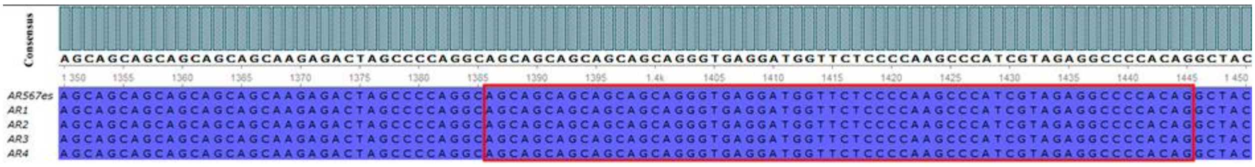

Figure S1: Sequence alignment of alternatively spliced AR variants. The red square indicates sequence of 60-AR_RNA used as a target RNA in the study.

3. *Oligonucleotides used in the study*

Table S2: Oligonucleotides.

Name	Sequence, from 5'to 3' termini
60-AR_RNA	agcagcagcagcagcagggugaggaugguucuccccaagcccaucguagaggccccacag
FAM-60-AR_RNA	[a]FAM-agcagcagcagcagcagggugaggaugguucuccccaagcccaucguagaggccccacag
T1	CCTCAGGCTAGCTACAACGACCTGCTGCT/[b]HEG/CGCGAGTGCG
T2	ccagguaacgaauggauuaaccauTTTTTTCACGGCCCCC
T3	GGGACCGGGCTTTTTTuggggaguuucccgacugg
T4	GCGTCGCGCGTTTTTTGGGGCCTCTAGGCTAGCTACAACGAGATGGGCT
T5	CGCGCGACGCGCCCGGTCCCGGGGGCCGTGCGCACTCGCG
[c]Dz1	CCTCAGGCTAGCTACAACGACCTGCTGCT
Dz2	GGGGCCTCTAGGCTAGCTACAACGAGATGGGCTT
62-RNA	augagcaagaaaaagucuuacauaaacagaacagguacauuugaauucacaaguuuuuucag
46-RNA	guacuugagcuccacuccgcagcgucugaaguugcuggacgcguac
FAM-62-RNA	FAM-augagcaagaaaaagucuuacauaaacagaacagguacauuugaauucacaaguuuuuucag
FAM-46-RNA	FAM-guacuugagcuccacuccgcagcgucugaaguugcuggacgcguac
AR_sense	GGGTAAGGGAAGTAGGTGGAA
AR_antisense	ACTGCGGCTGTGAAGGTT
GAPDH_sense	AGAGATGATGACCCTTTTGGC
GAPDH_antisense	CCATCACCATCTTCCAGGAGCG

[a]FAM – 6-fluorescein amidite, [b]HEG – hexoethylene glycol linker, [c] Dz – deoxyribozyme, [d] Uppercase letters indicate DNA chains, lowercase – RNA.

4. *Assembling of SDFS*

Assembling of SDFS was performed by heating the mix of T1–T5 oligonucleotides (2.5 μM for each strand) for 5 min at +95 °C, followed by cooling to +4 °C, with increments in temperature decrease of 0.5 °C/min. Annealing underwent in a buffer, containing 50 mM $MgCl_2$, 140 mM NaCl, 5 mM KCl, 50 mM HEPES, pH 7.4. The efficiency of the SDFS assembling process was checked using agarose gel-electrophoresis in 2% gel prepared on 1x TBE buffer (89 mM Tris, 89 mM H_3BO_3, 2 mM Na_2EDTA, pH 8.0). The obtained results were visualized using the ChemiDoc Imaging System (Bio-Rad Laboratories Inc., California, USA) after staining with SYBR™ Gold Nucleic Acid Gel Stain (Figure 1E).

5. *Cleavage of FAM-60-AR_RNA assay*

For proving that Dz1, Dz2 retained their functions in the composition of SDFS, both Dz1 and Dz2, as well as complete assembled SDFS were incubated in the presence of the FAM-60-AR_RNA target molecule at 37 °C in the reaction buffer (2 mM $MgCl_2$, 140 mM NaCl, 5 mM KCl, 50 mM HEPES, 0.25% DMSO, pH 7.4) for 6 h. The concentration of all oligonucleotides in the reaction mix, including 60-AR_RNA, was 100 nM. Then, the samples were taken out and put into the denaturing gel-loading buffer (8 M urea in 1x TBE). After that collected probes were analyzed by denaturing PAGE (17.5%, 7 M urea) at 80 V for 150 min and the results of cleavage are presented in Figure S2.

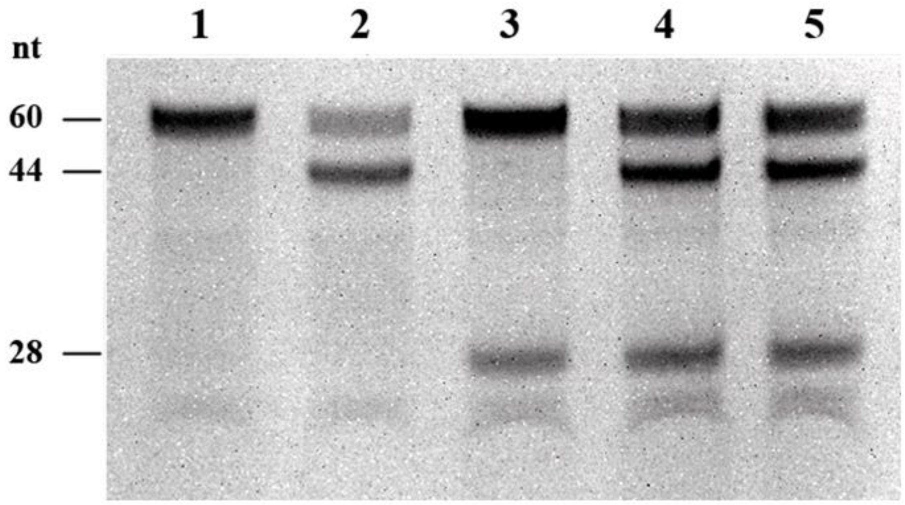

Figure S2: Assay of Dz1 and Dz2 cleavage activity. Lane 1: Single FAM-60-AR_RNA after 6 h of incubation. Lane 2: FAM-60-AR_RNA incubated with Dz1. Lane 3: FAM-60-AR_RNA incubated with Dz2. Lane 4: FAM-60-AR_RNA incubated with both Dz1 and Dz2. Lane 5: FAM-60-AR_RNA incubated with assembled SDFS.

The cleavage activity assay of SDFS and its unassembled tiles (Figure 2D) were performed using the same parameters of incubation and electrophoresis. One of the samples containing assembled SDFS and FAM-60-AR_RNA was incubated in the buffer described above with additional 25 μM of the malachite green dye. Percentages of the cleaved products were calculated from the obtained images. An efficiency of the SDFS catalytic activity against the FAM-labeled 60-AR_RNA was evaluated by electrophoresis followed by densitometry analysis of bands (ImageLab™ 6.0.1, Bio-Rad Laboratories Inc., California, USA).

6. *Fluorescence of SDFS in presence of 60-AR_RNA measurements*

SDFS was incubated in the presence of 60-AR_RNA at different temperatures (37 °C, 25 °C, or 4 °C) in the reaction buffer (2 mM $MgCl_2$, 140 mM NaCl, 5 mM KCl, 50 mM HEPES, 0.25% DMSO, pH 7.4) for 6 h or 24 h. The concentration of all oligonucleotides in the reaction mix, including 60-AR_RNA, was 100 nM, and the concentration of the malachite green dye was 25 μM. Then, samples were withdrawn and cooled for 1 h at +4 °C. After cooling, samples were removed, and the fluorescent signal was measured with an Agilent Cary Eclipse Fluorescence Spectrophotometer (Santa Clara, California, United States) at 648 nm wavelength (excitation wavelength at 610 nm). The data of three independent experiments were averaged and plotted using OriginPro 2015.

After the incubation with 60-AR_RNA for 6 h SDFS showed a significant fluorescent signal only for a sample that was incubated at 37 °C and demonstrated a LOD of about 1.6 nM (Figure S3), though after 24 h of incubation samples at 25 °C also showed a fluorescence signal.

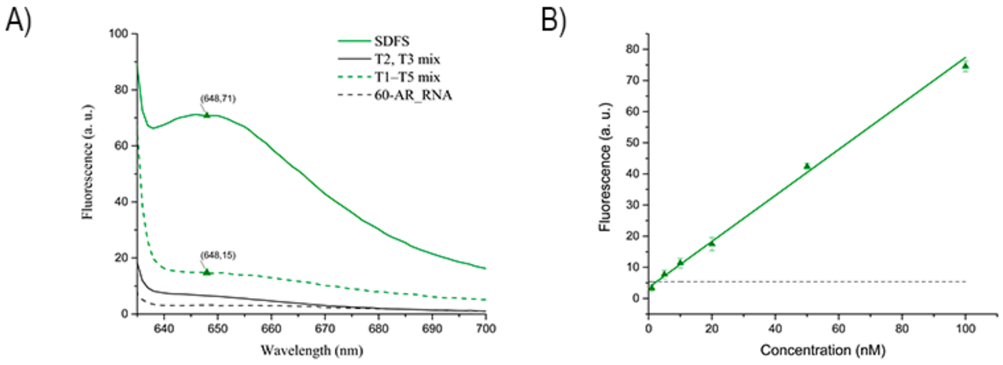

Figure S3: The SDFS functional activity after 6 h of incubation at 37 °C. A: emission spectra of assembled SDFS (green line), T1–T5 chains mix without annealing (green dotted line), aptamer sample containing chains T2 and T3 (black dotted line) and a control sample with 60-AR_RNA (black line) after 6 h of incubation. B: limit of detection for SDFS. Each dot is an average of triplicate values of fluorescence for samples with different concentrations of 60-

AR_RNA; standard deviations are shown. The green line is a linear regression line for these dots. The dashed indicates the threshold value of fluorescence of buffer containing malachite green.

7. *Limit of 60-AR_RNA detection for SDFS*

The same set of strands was incubated for determination of the limit of 60-AR_RNA detection (LOD) for 100 nM SDFS in the same buffer (2 mM $MgCl_2$, 140 mM NaCl, 5 mM KCl, 50 mM HEPES, 0.25% DMSO, pH 7.4) in the presence of 25 μM malachite green dye and different concentrations of 60-AR_RNA (1 nM, 5 nM, 10 nM, 20 nM, 50 nM, and 100 nM). After 6 h or 24 h of incubation and cooling, samples were removed, and the fluorescent signal was measured with an Agilent Cary Eclipse Fluorescence Spectrophotometer (Santa Clara, California, United States) at 648 nm wavelength (excitation wavelength at 610 nm). The data of three independent experiments were plotted using OriginPro 2015. The LOD was calculated as the concentration of 60-AR_RNA producing fluorescence, that is 3 standard deviations above the background.

8. *Selectivity of SDFS*

The selectivity of SDFS was tested with two random RNA sequences (Table S2, 62-RNA and 46-RNA). The fluorescence of such samples was measured, as described in chapter 7 and did not exceed the threshold value (data not shown). Each sample contained 1 μM RNA and was incubated for 6 h in a buffer (2 mM $MgCl_2$, 140 mM NaCl, 5 mM KCl, 50 mM HEPES, 0.25% DMSO, pH 7.4) with 100 nM SDFS and 25 μM malachite green dye. Verification of cleavage for samples, containing 5'-FAM labeled 62-RNA and 46-RNA (Table S2, FAM-62-RNA and FAM-46-RNA) instead of 62-RNA and 46-RNA was performed as described in chapter 5 (Figure S4).

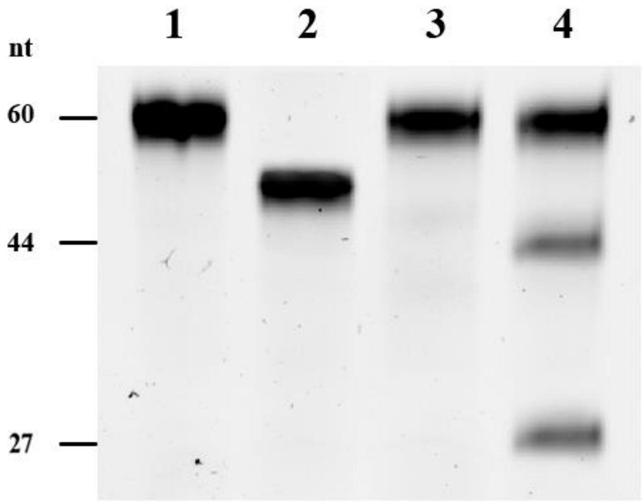

Figure S4: Selectivity of SDFS visualized by PAGE. Lane 1: FAM-62-RNA incubated with SDFS. Lane 2: FAM-46-RNA incubated with SDFS. Lane 3: FAM-60-AR_RNA incubated without SDFS. Lane 4: FAM-60-AR_RNA incubated with SDFS.

9. *Total RNA extraction*

Total RNA fraction was extracted from 1.5 million of both HDPC and HeLa cells in different passages (2, 3, 4, 5, 7) by ExtractRNA reagent following the manufacturer's protocol. The concentration of the obtained total RNA was measured using a NanoPhotometer® NP80 (Implen, München, Germany) after diluting in DNAse/RNAse-free water.

To verify AR expression in HDPC cells reverse transcription of extracted RNA and PCR using specific primers (AR_sense, AR_antisense) [2] were provided. GAPDH gene (primers are GAPDH_sense and GAPDH_antisense) was used as a normalization gene (Figure S5).

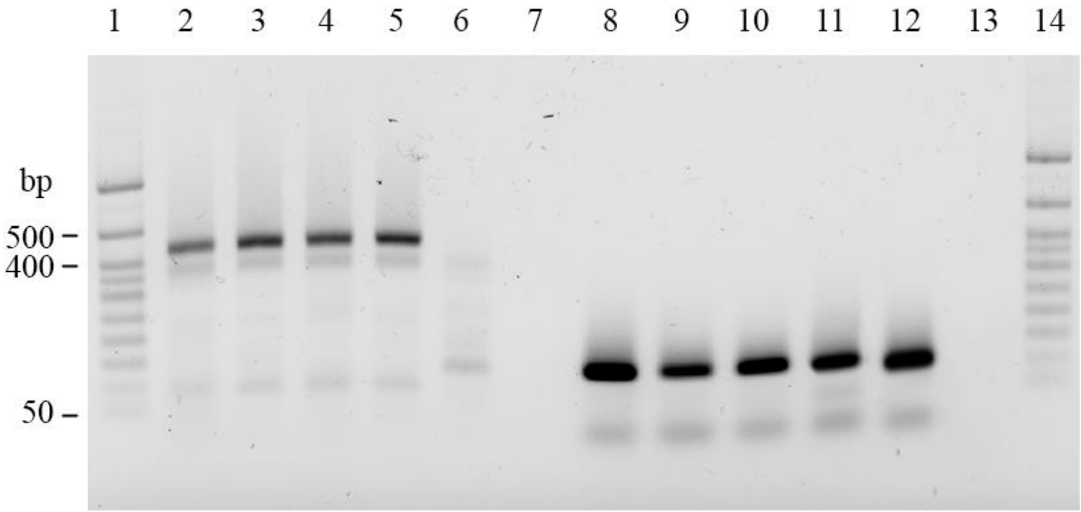

Figure S5: AR expression in HDPC cells visualized by agarose gel electrophoresis. Lane 1, 14: DNA ladder. Lane 2–7: Products of AR gene PCR for HDPC in passages 2, 3, 4, 5, 7 and negative control, respectively. Lane 8–13: Products of GAPDH gene PCR for HDPC in passages 2, 3, 4, 5, 7 and negative control, respectively.

According to the AR expression data, in experiments described in chapter 9 total RNA from 2 passage of both HDPC and HeLa cells was used.

10. *Fluorescence of SDFS in presence of HDPC total RNA measurements*

SDFS was incubated in the presence of 10 μg HDPC or HeLa total RNA at 37 °C in the reaction buffer (2 mM $MgCl_2$, 140 mM NaCl, 5 mM KCl, 50 mM HEPES, 0.25% DMSO, pH 7.4) for 24 h. The concentration of SDFS was 100 nM, and the concentration of malachite green dye was 25 μM. Then, samples were taken out and cooled for 1 h at +4 °C. After cooling the samples were removed, and the fluorescent signal was measured with an Agilent Cary Eclipse Fluorescence Spectrophotometer (Santa Clara, California, United States) at 648 nm wavelength (excitation wavelength at 610 nm). The data of three independent experiments were averaged and plotted using OriginPro 2015.

References:

1. Wach, S.; Taubert, H.; Cronauer, M. *World J. Urol.*, **2020**, *38(3)*, 647–656 .
2. Richeti, F.; Kochi, C.; Rocha, M.N.; Sant'anna Corrêa, C.; Lazzarini, R.; Guazzelli, R.M.; Mendonça, R.F.; Melo, M.R.; Longui, C.A. *Genet. Mol. Res.*, **2013**, *12*, 1834–1840.
